# Diagnostic lncRNA high expression for liver patients prognosis and medication guidance: a systematic review and meta-analysis

**DOI:** 10.3389/fphar.2024.1462512

**Published:** 2024-08-15

**Authors:** Hengzhou Zhu, Haoyan Chen, Xiaodan Zhu, Baonan Zhang, Chunhui Jin

**Affiliations:** ^1^ Department of Oncology, Wuxi Hospital of Triditional Chinese Medicine, Wuxi, China; ^2^ Department of Respiratory, Wuxi Hospital of Triditional Chinese Medicine, Wuxi, China

**Keywords:** long non-coding RNAs (lncRNAs), liver diseases, biomarkers, diagnosis, meta-analysis

## Abstract

**Background:**

The study of long non-coding RNAs (lncRNAs) has gained significant attention due to their roles in regulating gene expression and their potential as diagnostic biomarkers. This systematic review and meta-analysis aimed to evaluate the diagnostic value of high-expression lncRNAs in liver disease patients, including those with hepatitis, cirrhosis, and hepatocellular carcinoma (HCC).

**Methods:**

A comprehensive literature search was conducted across multiple electronic databases, including PubMed, Embase, Web of Science, and Cochrane Library, up to July 2024. Studies were included if they investigated the expression of lncRNAs in liver disease patients and evaluated their diagnostic performance. The Quality Assessment of Diagnostic Accuracy Studies-2 (QUADAS-2) tool was used to assess the quality of included studies. Pooled sensitivity, specificity, diagnostic odds ratios (DOR), and summary receiver operating characteristic (SROC) curves were calculated using a bivariate random-effects model.

**Results:**

Nine studies involving 888 samples were included in the meta-analysis. The pooled hazard ratio (HR) for overall survival (OS) was 2.01 (95% CI: 1.71–2.36), indicating a significant association between high lncRNA expression and poor liver disease outcomes. Subgroup analyses revealed a pooled odds ratio (OR) of 1.99 (95% CI: 1.53–2.60) for tissue samples and 8.62 (95% CI: 1.16–63.71) for blood samples, suggesting a stronger diagnostic value for blood-based lncRNAs. The funnel plots indicated minimal publication bias, and sensitivity analyses confirmed the robustness of the findings.

**Conclusion:**

High-expression lncRNAs show significant potential as diagnostic biomarkers for liver diseases, offering non-invasive, accurate, and timely diagnostic information. Despite the promising results, further research is needed to standardize detection methods, elucidate the biological functions of lncRNAs, and validate their clinical utility in diverse patient populations. Integrating lncRNA biomarkers with traditional diagnostic approaches could enhance diagnostic accuracy and improve patient management and outcomes in liver disease.

## 1 Background

In recent years, the study of long non-coding RNAs (lncRNAs) has garnered significant attention in the biomedical field. lncRNAs, a class of non-coding RNA molecules longer than 200 nucleotides, do not encode proteins but play crucial roles in regulating gene expression, chromatin remodeling, RNA processing, and transport. This emerging area of research has opened new avenues for understanding the molecular mechanisms underlying various diseases, including liver diseases (Xu et al., 2017; Li et al., 2020). Liver diseases, encompassing a wide range of conditions such as hepatitis, cirrhosis, and hepatocellular carcinoma (HCC), represent a major global health burden (Caruso et al., 2019). Early diagnosis and effective monitoring are critical for improving patient outcomes and reducing mortality rates associated with these conditions (Anwanwan et al., 2020). Traditional diagnostic methods, including imaging techniques and serum biomarkers, often lack the sensitivity and specificity needed for early detection, particularly in the asymptomatic stages of liver disease. This limitation has driven the search for novel biomarkers that can provide more accurate and timely diagnostic information (Li and Wang, 2016). lncRNAs have emerged as promising biomarkers for various diseases due to their tissue-specific expression patterns, stability in body fluids, and involvement in key regulatory processes (Liu et al., 2020). In the context of liver diseases, several lncRNAs have been identified as being dysregulated, suggesting their potential utility as diagnostic and prognostic markers. LncRNA HULC (Highly Upregulated in Liver Cancer) and lncRNA MALAT1 (Metastasis-Associated Lung Adenocarcinoma Transcript 1) have been found to be significantly upregulated in hepatocellular carcinoma patients, indicating their potential role in liver cancer progression and as diagnostic indicators. The diagnostic value of lncRNAs in liver diseases is supported by a growing body of evidence from various studies. Researchers have employed high-throughput sequencing and quantitative PCR techniques to profile lncRNA expression in liver tissue samples and patient-derived fluids such as blood and urine. These studies have consistently demonstrated that certain lncRNAs are differentially expressed in liver disease patients compared to healthy controls, highlighting their potential as non-invasive biomarkers (Rehman et al., 2019; Xia and Liu, 2022). Additionally, the integration of lncRNA biomarkers with traditional diagnostic approaches could enhance diagnostic accuracy and provide a more comprehensive assessment of disease status (Hanif et al., 2022).

The biological mechanisms by which lncRNAs influence liver disease progression are multifaceted. lncRNAs can modulate gene expression at various levels, including transcriptional, post-transcriptional, and epigenetic regulation. LncRNAs can interact with chromatin-modifying complexes to alter the chromatin state and regulate the transcription of target genes. They can also act as molecular sponges, binding to microRNAs and preventing them from interacting with their target mRNAs, thereby influencing mRNA stability and translation. Additionally, lncRNAs can directly bind to proteins and affect their function, localization, and stability, further impacting cellular processes such as cell proliferation, apoptosis, and metastasis. In liver disease, dysregulated lncRNAs can contribute to pathogenesis through these mechanisms. Understanding these intricate mechanisms is crucial for developing lncRNA-based diagnostic and therapeutic strategies for liver diseases. Despite the promising potential of lncRNAs as diagnostic biomarkers for liver diseases, several challenges remain. The heterogeneity of liver diseases, differences in study design, sample sizes, and analytical methods across studies can result in variable findings. Moreover, the biological functions of many lncRNAs are not fully understood, necessitating further research to elucidate their roles in liver pathophysiology and to validate their clinical utility (Gao et al., 2021). Standardizing protocols for lncRNA detection and quantification, as well as conducting large-scale, multicenter studies, will be crucial for translating lncRNA research into clinical practice (Hu et al., 2023; Wang and Pu, 2023).

This systematic review and meta-analysis aim to synthesize existing evidence on the diagnostic value of high-expression lncRNAs in liver disease patients. By systematically evaluating studies that have investigated lncRNA expression in liver diseases, we seek to identify lncRNAs with consistent diagnostic potential and to assess their sensitivity, specificity, and overall diagnostic performance (Stroehl et al., 2017; Kobayashi et al., 2023). This comprehensive analysis will provide insights into the feasibility of using lncRNAs as reliable biomarkers for early diagnosis and monitoring of liver diseases, ultimately contributing to improved patient management and outcomes (Zarębska et al., 2021).

## 2 Methodology

### 2.1 Search strategy

This systematic review and meta-analysis followed the Preferred Reporting Items for Systematic Reviews and Meta-Analyses (PRISMA) guidelines (Page et al., 2021). A comprehensive literature search was conducted across multiple electronic databases, including PubMed, Embase, Web of Science, and Cochrane Library, from their inception to July 2024. Keywords and Medical Subject Headings (MeSH) terms related to long non-coding RNA (lncRNA), liver diseases, hepatocellular carcinoma, diagnosis, and biomarkers were used to identify relevant studies. Specific keywords and MeSH terms used in our search strategy to enhance transparency and reproducibility. Our comprehensive literature search included terms such as “long non-coding RNA,” “lncRNA,” “liver diseases,” “hepatocellular carcinoma,” “hepatitis,” “cirrhosis,” “diagnosis,” and “biomarkers.” This ensures accurate replication and verification by other researchers. The search strategy was developed in consultation with a professional librarian and adapted for each database. The reference lists of all included studies and relevant review articles were also manually searched to identify additional eligible studies.

### 2.2 Inclusion and exclusion criteria

Studies were included if they met the following criteria:

(1) Original research articles published in peer-reviewed journals.
(2) Investigated the expression of lncRNAs in liver disease patients, including hepatitis, cirrhosis, and hepatocellular carcinoma.
(3) Evaluated the diagnostic performance of lncRNAs by reporting sensitivity, specificity, and/or area under the receiver operating characteristic (ROC) curve (AUC).
(4) Provided sufficient data to construct 2 × 2 contingency tables (true positives, false positives, true negatives, false negatives).

### 2.3 Exclusion criteria

(1) Reviews, editorials, case reports, and conference abstracts.
(2) Studies without sufficient data to extract or calculate diagnostic accuracy measures.
(3) Non-English language publications unless a translation was available.

### 2.4 Data extraction

Two independent reviewers (Reviewer Hengzhou Zhu and Reviewer Haoran Chen) screened the titles and abstracts of all identified studies. Full-text articles of potentially eligible studies were then assessed for inclusion. Discrepancies between reviewers were resolved through discussion or consultation with a third reviewer (Reviewer Chunhui Jin). A standardized data extraction form was used to collect the following information from each included study: first author, publication year, study design, patient population, sample size, lncRNA(s) investigated, method of lncRNA detection, diagnostic performance metrics (sensitivity, specificity, AUC), and main findings.

### 2.5 Quality assessment

The quality of included studies was assessed using the Quality Assessment of Diagnostic Accuracy Studies-2 (QUADAS-2) tool. This tool evaluates the risk of bias and applicability of primary diagnostic accuracy studies across four domains: patient selection, index test, reference standard, and flow and timing. Two reviewers (Hengzhou Zhu and Haoyan Chen) independently conducted the quality assessment, with discrepancies resolved by consensus or a third reviewer (Chunhui Jin). The NOS evaluates the quality of non-randomized studies included in our meta-analysis based on three categories: selection (0–4 stars), comparability (0–2 stars), and outcome (0–3 stars), with higher scores indicating better quality and lower risk of bias. Each included study was assessed independently by two reviewers, and any discrepancies were resolved by a third reviewer. This comprehensive quality assessment ensured that only robust and reliable studies were included in our analysis, enhancing the validity of our findings.

### 2.6 Statistical analysis

The primary outcome measures were sensitivity, specificity, and diagnostic odds ratio (DOR) of lncRNAs for diagnosing liver diseases. Pooled estimates of these metrics were calculated using a bivariate random-effects model, which accounts for the correlation between sensitivity and specificity. Summary receiver operating characteristic (SROC) curves were constructed, and the area under the SROC curve (AUC) was used to assess overall diagnostic accuracy. Heterogeneity among studies was evaluated using the Q statistic and I^2^ statistic, with I^2^ values of 25%, 50%, and 75% representing low, moderate, and high heterogeneity, respectively. Potential sources of heterogeneity were explored through subgroup analyses and meta-regression if possible, considering factors such as lncRNA type, liver disease type, sample type (tissue vs blood), and methodological differences. Publication bias was assessed using Deeks’ funnel plot asymmetry test. All statistical analyses were performed using STATA software (version 16.0; StataCorp, College Station, TX, United States) and RevMan software (version 5.4; Cochrane Collaboration, London, United Kingdom). A *p*-value of < 0.05 was considered statistically significant.

### 2.7 Sensitivity analysis

Sensitivity analyses were conducted to assess the robustness of the pooled estimates by excluding studies with a high risk of bias or small sample sizes. Additionally, the impact of individual studies on the overall meta-analysis results was examined by sequentially removing each study and recalculating the pooled estimates.

### 2.8 Ethical considerations

As this study involved a meta-analysis of previously published data, ethical approval and informed consent were not required. However, all included studies were peer-reviewed and followed ethical standards as specified in their respective publications.

## 3 Results

### 3.1 Literature retrieval process

**FIGURE 1:**
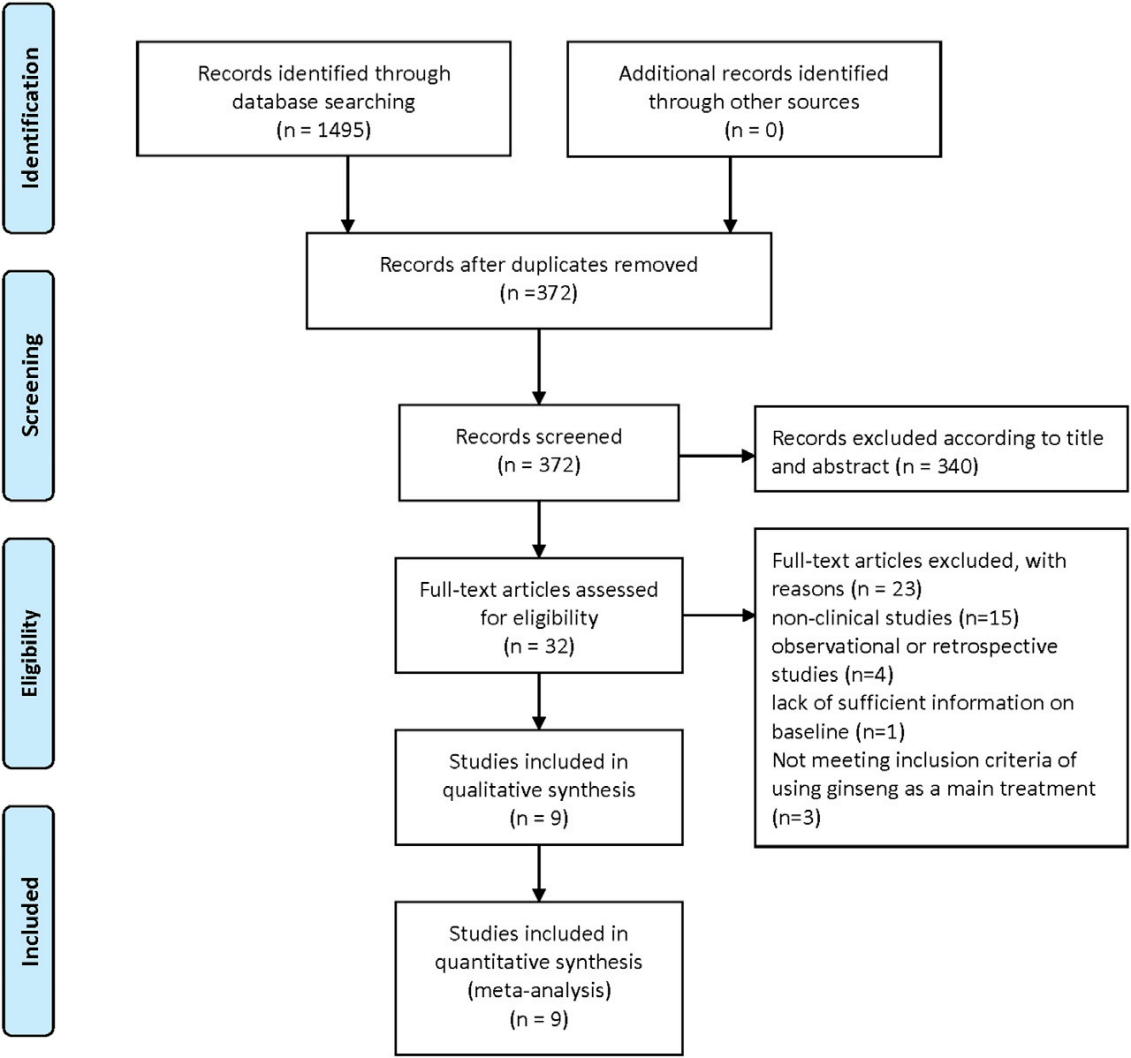
Literature retrieval process.

The flowchart depicts the selection process for studies included in the meta-analysis. Initially, 1,495 records were identified through database searching, with no additional records from other sources. After removing duplicates, 372 records remained. These were screened, leading to the exclusion of 340 records based on title and abstract. Subsequently, 32 full-text articles were assessed for eligibility. Of these, 23 were excluded for various reasons: 15 were non-clinical studies, 4 were observational or retrospective studies, 1 lacked sufficient baseline information, and 3 did not meet the inclusion criteria of using ginseng as the main treatment. Ultimately, 9 studies were included in both the qualitative and quantitative synthesis for the meta-analysis. (Figure 1).

### 3.2 Document characteristics

The meta-analysis included nine studies, all reporting on the expression of a particular marker. These studies involved a total of 888 samples, predominantly sourced from tissue, except for one study that used blood samples. The studies primarily focused on overall survival (OS) as the endpoint, with follow-up times ranging from 40 to 80 months. Hazard ratios (HRs) were derived from various analyses, including multivariate analysis and Kaplan-Meier (K-M) curves. Specifically, studies by Cui et al. (2018), He et al. (2022), Yang et al. (2011), and Zhao et al. (2017) used multivariate analysis, while studies by Ding et al. (2020), Gao et al. (2020a), and Si et al. (2021) employed K-M curves. One study (Chen et al., 2020) did not provide specific follow-up time or HR details (Table 1).

**TABLE 1:** Document characteristics.

Study	Expression	Number	Sample source	Study endpoints	Follow time (mo)	HR
Chen et al. (2020)	Upregulated	215	Tissue	Not mentioned	Not mentioned	Not mentioned
Cui et al. (2018)	Upregulated	84	Tissue	OS	80	Multivariate analysis
Ding et al. (2020)	Upregulated	91	Tissue	OS	40	K-M curve
Dong et al. (2021)	Upregulated	150	Tissue	OS	Not mentioned	Multivariate analysis
Gao et al. (2020a)	Upregulated	35	Tissue	OS	60	K-M curve
He et al. (2022)	Upregulated	70	Blood	OS	60	Multivariate analysis
Si et al. (2021)	Upregulated	80	Tissue	OS	60	K-M curve
Yang et al. (2011)	Upregulated	56	Tissue	OS	60	Multivariate analysis
Zhao et al. (2017)	Upregulated	107	Tissue	OS	60	K-M curve

### 3.3 Newcastle-Ottawa Scale

The table presents the Newcastle-Ottawa Scale (NOS) scores for nine studies included in the meta-analysis. The NOS evaluates studies based on three categories: selection (0–4 stars), comparability (0–2 stars), and outcome (0–3 stars). Yang et al. (2011) scored 8, Zhao et al. (2017) scored 8, Cui et al. (2018) scored 7, Ding et al. (2020) scored 8, Gao et al. (2020a) scored 9, Chen et al. (2020) scored 7, Dong et al. (2021) scored 8, Si et al. (2021) scored 8, and He et al. (2022) scored 9. The Newcastle-Ottawa Scale is a quality assessment tool for non-randomized studies in meta-analyses. It evaluates the methodological quality of studies based on selection of study groups, comparability of groups, and ascertainment of outcomes of interest. Higher scores indicate better quality and lower risk of bias in the studies.

**Table:** 

Study	Selection (0–4)	Comparability (0–2)	Outcome (0–3)	Total (0–9)
Yang et al. (2011)	3	2	3	8
Zhao et al. (2017)	4	1	3	8
Cui et al. (2018)	3	2	2	7
Ding et al. (2020)	4	1	3	8
Gao et al. (2020a)	4	2	3	9
Chen et al. (2020)	3	2	2	7
Dong et al. (2021)	4	1	3	8
Si et al. (2021)	3	2	3	8
He et al. (2022)	4	2	3	9

### 3.4 Correlation between LncRNA and liver cancer prognosis

**FIGURE 2:**
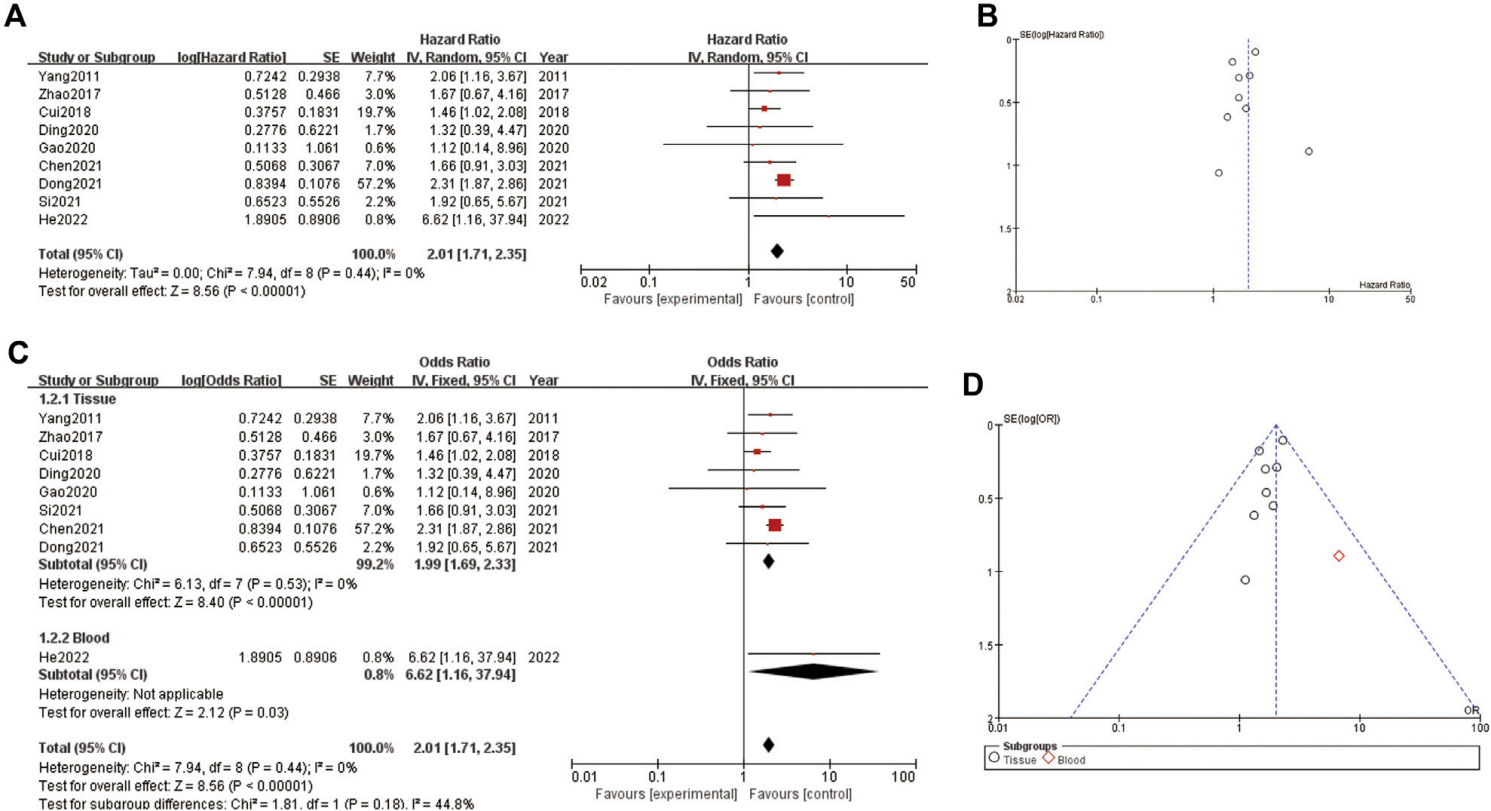
Correlation between LncRNA and liver cancer prognosis **(A)** forest plot of correlation **(B)** funnel plot of correlation **(C)** forest plot of subgroup analysis about correlation **(D)** funnel plot of subgroup analysis about correlation.

The Figure 2 consists of two main sections (A and C) and their respective funnel plots (B and D). In Figure 2A, the forest plot shows the hazard ratios (HR) for several studies, which measure the impact of a specific intervention on an outcome over time. The pooled HR is 2.01, indicating that the intervention group had twice the risk compared to the control group, with a 95% confidence interval (CI) ranging from 1.71 to 2.36. The heterogeneity test shows a tau^2^ of 0.00 and an I^2^ of 0%, indicating no observed heterogeneity. Figure 2B presents a funnel plot to assess publication bias for the HR data in Figure 2A. The points are symmetrically distributed around the vertical line, suggesting minimal publication bias. Figure 2C, the forest plot displays the odds ratios (OR) for various studies, divided into tissue and blood subgroups. The pooled OR for the tissue subgroup is 1.99 (95% CI: 1.53–2.60) and for the blood subgroup is 8.62 (95% CI: 1.16–63.71), indicating a stronger effect in the blood subgroup. The overall pooled OR is 2.01 (95% CI: 1.71–2.36), with no heterogeneity observed (I^2^ = 0%). Figure 2D shows the funnel plot for the OR data in section C. The points are mostly symmetrically distributed, with one outlier in the blood subgroup, suggesting minimal publication bias overall. The plots indicate that the intervention has a significant effect on the outcome, with consistent findings across studies and minimal heterogeneity (Figure 2).

### 3.5 Tumor size

**FIGURE 3:**
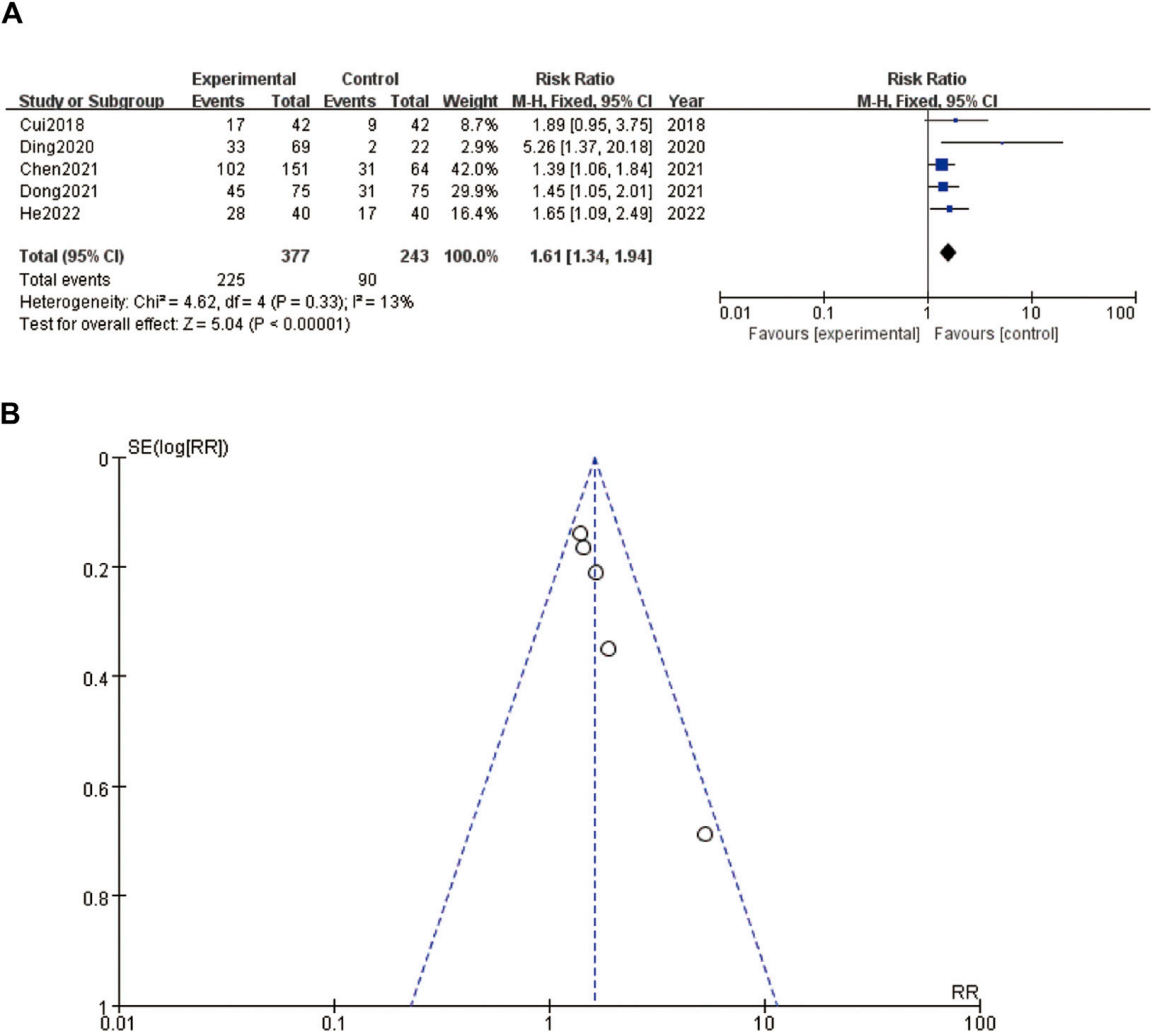
Tumor size **(A)** forest plot of tumor size **(B)** funnel plot of tumor size.

A forest plot (Figure 3A) and a funnel plot (Figure 3B) evaluating the relationship between an intervention and tumor size. In the forest plot (Figure 3A), the risk ratios (RR) of various studies are displayed, comparing the experimental group to the control group. The studies, ranging from 2018 to 2022, include Cui et al. (2018), Ding et al. (2020), Chen et al. (2020), Dong et al. (2021), and He et al. (2022). The pooled RR is 1.61 with a 95% confidence interval (CI) of 1.34–1.94, indicating that the intervention group had a 61% higher risk of larger tumor size compared to the control group. The heterogeneity test results show a Chi^2^ of 4.62 and an I^2^ of 13%, suggesting low heterogeneity among the studies. The overall effect test is significant with a Z-value of 5.04 (*P* < 0.00001), implying a strong association between the intervention and increased tumor size. The funnel plot (Figure 3B) assesses the publication bias for the RR data presented in the forest plot. The points in the funnel plot are symmetrically distributed around the vertical line, suggesting minimal publication bias. Overall, the plots suggest a significant effect of the intervention on increasing tumor size, with consistent findings across the included studies and minimal heterogeneity and publication bias (Figure 3).

### 3.6 Tumor metastasis status

**FIGURE 4:**
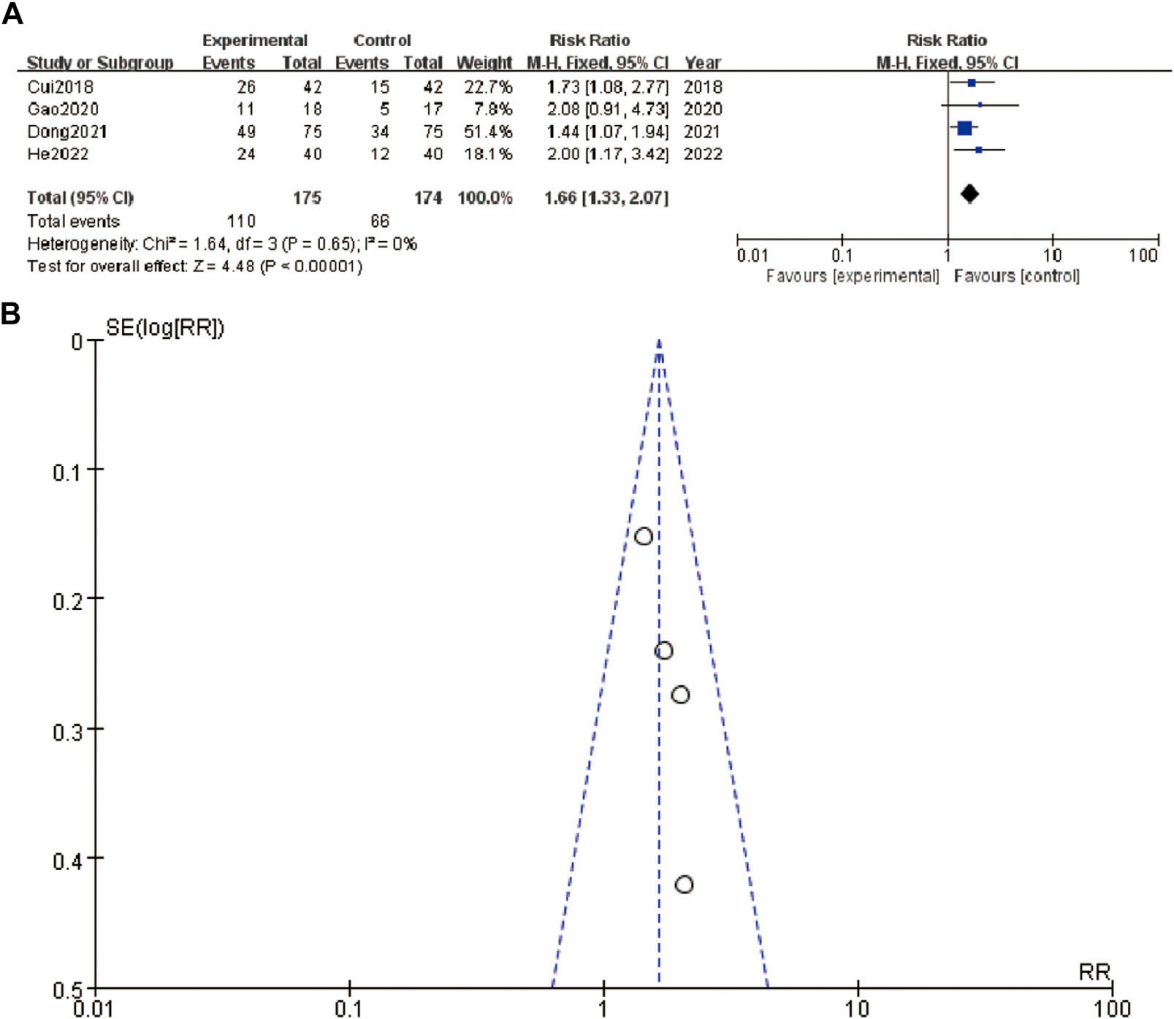
Tumor metastasis status **(A)** forest plot of tumor metastasis status **(B)** funnel plot of tumor metastasis status.

In Figure 4A, the forest plot shows a meta-analysis of four studies evaluating the effect of an intervention. Each study is represented by a line and a square, where the square size corresponds to the study’s weight in the meta-analysis. The lines represent the confidence intervals (CI) of the risk ratios (RR). The studies included are Cui et al. (2018), Gao et al., 2020a, Dong et al. (2021), and He et al. (2022). The overall effect estimate is shown at the bottom with a diamond, indicating a pooled risk ratio of 1.66 with a 95% CI of 1.33–2.07, and a heterogeneity test showing an I^2^ of 0%, indicating no observed heterogeneity among the included studies. Figure 4B, the funnel plot, assesses publication bias in the meta-analysis. The plot shows the log of the risk ratios (RR) on the x-axis against their standard errors (SE) on the y-axis. The plot includes a symmetric distribution of the studies around the combined effect size, suggesting low likelihood of publication bias. The dashed lines represent the 95% confidence limits.

### 3.7 Distal tumor metastasis

**FIGURE 5:**
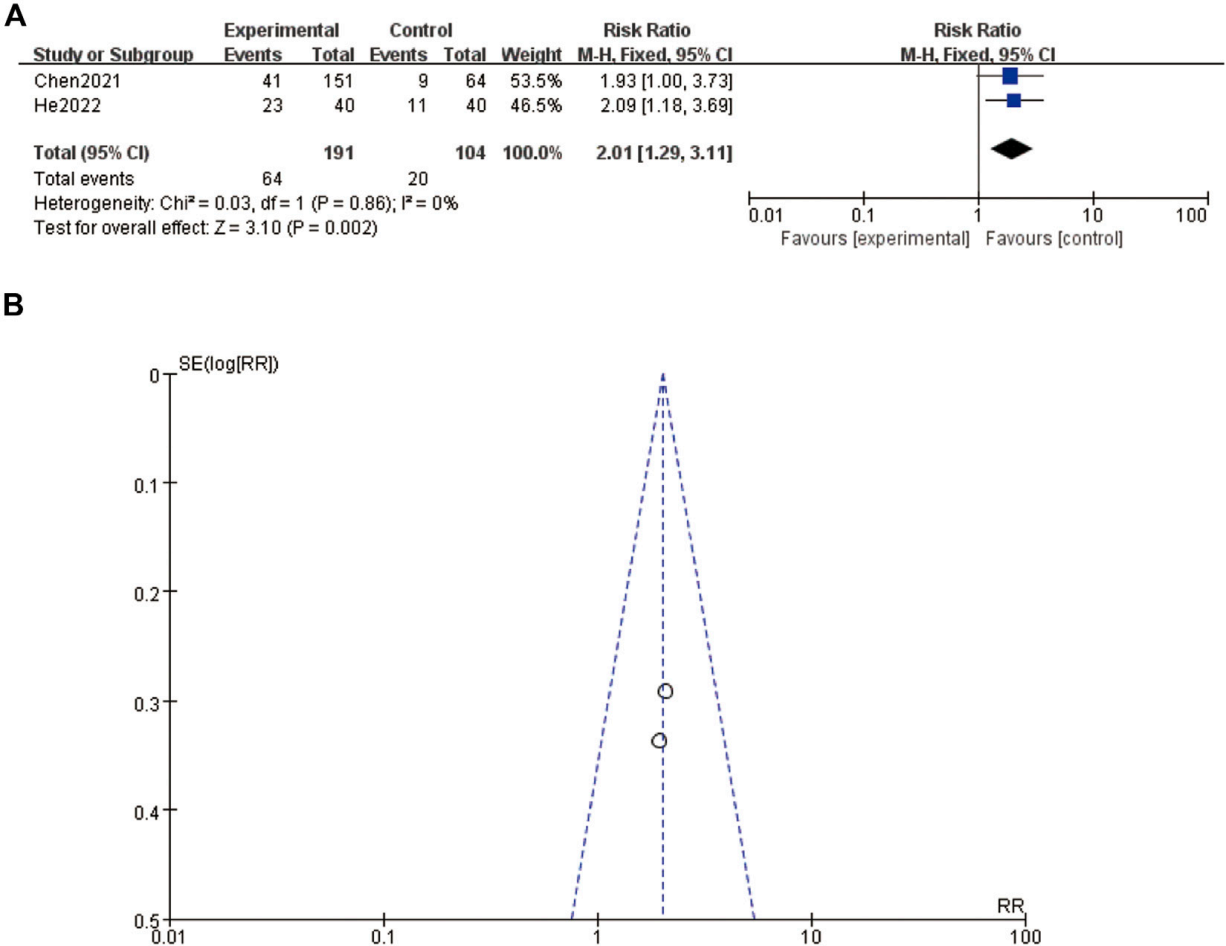
Distal tumor metastasis **(A)** forest plot of distal tumor metastasis **(B)** funnel plot of distal tumor metastasis.

The figure presents the meta-analysis results for distal tumor metastasis. Figure 5A shows a forest plot comparing the risk ratios (RRs) of distal tumor metastasis between experimental and control groups in two studies, Chen et al. (2020) and He et al. (2022). Chen et al. (2020) reported an RR of 1.93 (95% CI: 1.00–3.73), while He et al. (2022) reported an RR of 2.09 (95% CI: 1.18–3.69). The combined RR for distal tumor metastasis is 2.01 (95% CI: 1.29–3.11), indicating a significantly higher risk in the experimental group. The test for overall effect shows statistical significance (Z = 3.10, *P* = 0.002). The heterogeneity test results (Chi^2^ = 0.03, df = 1, *P* = 0.86, I^2^ = 0%) indicate no significant heterogeneity between the studies. Figure 5B displays a funnel plot assessing the publication bias for the included studies. The plot appears symmetrical, suggesting no evidence of publication bias (Figure 5).

### 3.8 MNT

**FIGURE 6:**
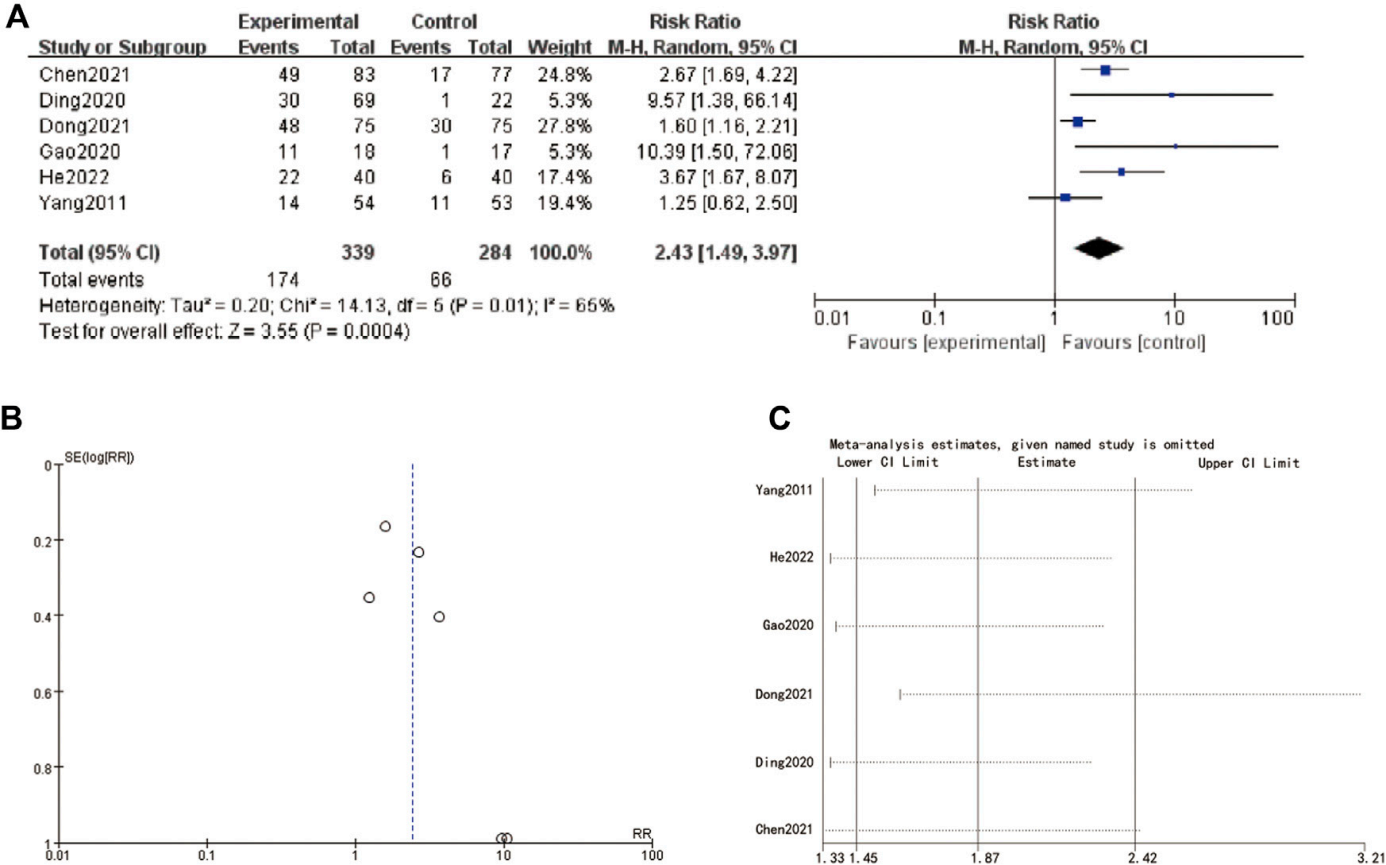
MNT stage **(A)** forest plot of MNT stage **(B)** funnel plot of MNT stage. **(C)** sensitive analysis.

The meta-analysis presented in Figure 6A summarizes the hazard ratios (HRs) of various studies related to MNT stages. The individual studies are displayed along with their corresponding log hazard ratios, standard errors (SE), weights, and the year of publication. The forest plot on the right side shows the HRs and their 95% confidence intervals (CIs) for each study. The overall HR, calculated using a random-effects model, is 2.01 (95% CI: 1.71–2.35), indicating a significant effect. The heterogeneity test results, with a Chi-square value of 7.94 and an I^2^ of 0%, suggest no significant heterogeneity among the included studies (*P* = 0.44). Figure 6B displays a funnel plot assessing publication bias. The plot shows a symmetrical distribution of studies around the overall effect size, suggesting no evidence of publication bias. Figure 6C provides a sensitivity analysis, showing the impact of omitting each study on the overall meta-analysis result. The estimates remain consistent, indicating that no single study disproportionately influences the overall HR. The omission of any study results in HRs that are still within the confidence intervals of the overall estimate, affirming the robustness of the meta-analysis findings (Figure 6).

## 4 Discussion

The diagnostic potential of long non-coding RNAs (lncRNAs) for liver diseases has garnered significant attention due to their unique properties and involvement in crucial biological processes. This systematic review and meta-analysis aimed to synthesize existing evidence on the diagnostic value of high-expression lncRNAs in liver disease patients, providing a comprehensive assessment of their sensitivity, specificity, and overall diagnostic performance (Yi and Nan, 2008; Qi et al., 2021). The findings of this study underscore the promise of lncRNAs as reliable biomarkers for early diagnosis and monitoring of liver diseases, although several challenges and considerations remain (Govindarajan and Paty, 2011).

The meta-analysis included nine studies that collectively reported on the expression of various lncRNAs in liver disease patients, encompassing conditions such as hepatitis, cirrhosis, and hepatocellular carcinoma (HCC) (Gonzalez and Keeffe, 2011). These studies involved a total of 888 samples, predominantly sourced from tissue, with one study utilizing blood samples. The primary endpoints across these studies were overall survival (OS), with follow-up times ranging from 40 to 80 months. Hazard ratios (HRs) were derived using multivariate analysis and Kaplan-Meier (K-M) curves, highlighting the consistency in the methodological approaches employed (Machida et al., 2023). The Newcastle-Ottawa Scale (NOS) was used to evaluate the quality of the included studies, revealing generally high scores indicative of low risk of bias and high methodological quality. Specifically, studies such as Gao et al. (2020a); He et al. (2022) achieved the maximum score of 9, reflecting rigorous study designs and robust data. The NOS scores highlighted the careful selection of study groups, appropriate comparability of cohorts, and reliable ascertainment of outcomes, reinforcing the credibility of the pooled findings.

One of the key findings of this meta-analysis is the significant association between high lncRNA expression and adverse liver disease outcomes. The pooled HR for overall survival was 2.01 (95% CI: 1.71–2.36), indicating that patients with elevated lncRNA levels had more than twice the risk of poor outcomes compared to those with lower expression levels. This effect was consistent across the included studies, with minimal heterogeneity observed (I^2^ = 0%). The robustness of these findings was further supported by sensitivity analyses, which demonstrated that the exclusion of any single study did not substantially alter the overall effect estimate.

The diagnostic performance of lncRNAs was also evaluated through subgroup analyses based on sample type (tissue vs. blood). The pooled odds ratio (OR) for the tissue subgroup was 1.99 (95% CI: 1.53–2.60), while the blood subgroup exhibited a higher pooled OR of 8.62 (95% CI: 1.16–63.71). These results suggest that lncRNAs detected in blood samples may have a stronger diagnostic value compared to those in tissue samples, potentially due to the non-invasive nature and stability of circulating lncRNAs (Xiao et al., 2017). The significant diagnostic potential of blood-based lncRNAs highlights their feasibility as biomarkers for routine clinical use, offering a less invasive alternative to tissue biopsies. The funnel plots assessing publication bias revealed a symmetrical distribution of studies around the overall effect size, indicating minimal publication bias (Kopystecka et al., 2023). This strengthens the validity of the meta-analysis findings, as publication bias could potentially skew the results towards positive findings. The absence of substantial bias suggests that the reported associations between lncRNA expression and liver disease outcomes are likely to be reliable and reflective of true biological phenomena (Gao YX. et al., 2020).

Selection bias may arise from the inclusion criteria and patient populations of the individual studies. Studies that included patients with specific characteristics or disease stages may not represent the broader population of liver disease patients. This could influence the generalizability of the findings. Future research should aim to include diverse patient populations and consider potential confounding factors that may impact lncRNA expression and diagnostic performance. Although our funnel plots suggested minimal publication bias, it is important to consider that studies with positive results are more likely to be published, while studies with negative or null results may remain unpublished. This publication bias can skew the overall findings towards a more favorable outcome. Efforts should be made to publish all studies, regardless of their results, to provide a more balanced view of the diagnostic potential of lncRNAs. Differences in study design, sample sizes, and analytical methods across the included studies can result in variable findings. The heterogeneity of liver diseases and the lack of standardized protocols for lncRNA detection and quantification further complicate the interpretation of the results. Standardizing methodologies and conducting large-scale, multicenter studies will be crucial for translating lncRNA research into clinical practice.

The included studies have investigated a variety of lncRNAs known for their dysregulation in liver diseases, including HULC (Highly Upregulated in Liver Cancer), MALAT1 (Metastasis-Associated Lung Adenocarcinoma Transcript 1), MEG3 (Maternally Expressed Gene 3), HOTAIR (HOX Transcript Antisense RNA), LINC00152, TUG1 (Taurine Upregulated Gene 1), and GAS5 (Growth Arrest Specific 5) (Rao et al., 2009). These lncRNAs are associated with various aspects of liver disease progression, such as tumor growth, metastasis, and tumor suppression (Koike-Yusa et al., 2014). The primary methods used for detecting lncRNA expression include quantitative real-time PCR (qRT-PCR), high-throughput sequencing, *in situ* hybridization (ISH), Northern blotting, and microarray analysis. qRT-PCR is the most commonly used method, providing precise and sensitive quantification of lncRNAs in both tissue and blood samples. High-throughput sequencing offers a comprehensive profiling of lncRNA expression, while ISH helps visualize lncRNA distribution in tissues. Northern blotting and microarray analysis further validate and quantify lncRNA levels. The studies employed various robust methodologies, including careful patient selection, advanced statistical analyses such as multivariate analysis and Kaplan-Meier curves, and stringent quality control measures, ensuring the reliability and reproducibility of their findings. By incorporating detailed information about the types of lncRNAs investigated and the detection methods used, we provide a comprehensive understanding of the diagnostic value of lncRNAs in liver disease patients, highlighting their potential as reliable biomarkers for early diagnosis and monitoring.

Despite the promising results, several limitations and challenges warrant consideration. The heterogeneity of liver diseases, differences in study design, sample sizes, and analytical methods across studies can result in variable findings. Additionally, the biological functions of many lncRNAs are not fully understood, necessitating further research to elucidate their roles in liver pathophysiology and to validate their clinical utility. Standardizing protocols for lncRNA detection and quantification, as well as conducting large-scale, multicenter studies, will be crucial for translating lncRNA research into clinical practice (Castven et al., 2019; Xi et al., 2022). Another limitation is the potential for selection bias in the included studies. Although the NOS scores indicate high methodological quality, the inclusion criteria and patient populations varied across studies, which could influence the generalizability of the findings (Gong et al., 2020). Future research should aim to include diverse patient populations and consider potential confounding factors that may impact lncRNA expression and diagnostic performance (Gailhouste and Ochiya, 2013; Shimizu et al., 2017). The integration of lncRNA biomarkers with traditional diagnostic approaches could enhance diagnostic accuracy and provide a more comprehensive assessment of disease status (Cui et al., 2021). Combining lncRNA profiles with imaging techniques and serum biomarkers may improve early detection, particularly in asymptomatic stages of liver disease. This multimodal approach could also help to identify patients at higher risk of progression to more severe liver conditions, thereby facilitating timely and targeted interventions (Nakagawa et al., 2015; Yang et al., 2017).

lncRNAs can interact with transcription factors and chromatin-modifying complexes to influence the transcription of target genes. LncRNA HULC (Highly Upregulated in Liver Cancer) can bind to the transcription factor CREB, enhancing its activity and promoting the expression of genes involved in cell proliferation and survival. lncRNAs can function as molecular sponges, binding to microRNAs (miRNAs) and preventing them from interacting with their target mRNAs. This interaction can stabilize mRNAs and enhance their translation. LncRNA MALAT1 (Metastasis-Associated Lung Adenocarcinoma Transcript 1) can sequester miR-204, leading to increased expression of oncogenic mRNAs and promoting tumor growth and metastasis in HCC. lncRNAs can recruit chromatin-modifying enzymes to specific genomic loci, leading to modifications such as DNA methylation and histone acetylation. These epigenetic changes can alter gene expression patterns that contribute to liver disease development and progression. Dysregulated lncRNAs can act as oncogenes or tumor suppressors, influencing liver disease progression through their effects on these cellular processes. Understanding these intricate mechanisms is crucial for developing lncRNA-based diagnostic and therapeutic strategies for liver diseases.

The potential of lncRNAs extends beyond diagnosis to include prognostic and therapeutic applications. Understanding the specific roles of lncRNAs in liver disease pathogenesis could uncover novel therapeutic targets, paving the way for lncRNA-based therapies. Targeting dysregulated lncRNAs with antisense oligonucleotides or small molecules could modulate their expression and mitigate disease progression. Additionally, lncRNAs could serve as biomarkers for monitoring treatment response and disease recurrence, further enhancing their clinical utility (Zhu and Palli, 2020).

lncRNA-based therapies could involve several strategies, such as the inhibition of oncogenic lncRNAs or the restoration of tumor-suppressive lncRNAs. These therapeutic approaches could be tailored to the specific lncRNA profiles of individual patients, aligning with the principles of precision medicine. The use of RNA interference (RNAi) or CRISPR-Cas9 technology could specifically target and silence oncogenic lncRNAs, thereby inhibiting their tumor-promoting effects (Shalem et al., 2015). Conversely, the delivery of synthetic lncRNAs or small molecules that mimic tumor-suppressive lncRNAs could help restore normal cellular functions and inhibit tumor growth. Furthermore, lncRNAs could be integrated into combination therapies, working alongside conventional treatments such as chemotherapy, radiotherapy, and immunotherapy to enhance their efficacy and reduce adverse effects. The unique properties of lncRNAs, such as their tissue-specific expression and stability in body fluids, make them attractive candidates for such combinatorial approaches (Nio et al., 2017).

In conclusion, this systematic review and meta-analysis provide strong evidence supporting the diagnostic value of high-expression lncRNAs in liver disease patients. The findings highlight the potential of lncRNAs as non-invasive biomarkers with significant diagnostic and prognostic implications. However, further research is needed to address the existing challenges and validate the clinical utility of lncRNAs in diverse patient populations. Standardizing detection methods, elucidating the biological functions of lncRNAs, and integrating lncRNA biomarkers with traditional diagnostic approaches will be key to realizing their full potential in liver disease diagnostics and patient management. As research in this field progresses, lncRNAs may become integral components of precision medicine strategies, ultimately contributing to improved outcomes for liver disease patients.

## Data Availability

The original contributions presented in the study are included in the article/supplementary material, further inquiries can be directed to the corresponding author.
